# Sarcoidosis Following Hematopoietic Stem Cell Transplantation: Clinical Characteristics and HLA Associations

**DOI:** 10.3389/fimmu.2021.746996

**Published:** 2021-10-07

**Authors:** Rebecca Isabel Wurm-Kuczera, Judith Buentzel, Julia Felicitas Leni Koenig, Tobias Legler, Jan-Jakob Valk, Justin Hasenkamp, Wolfram Jung, Jan-Gerd Rademacher, Peter Korsten, Gerald Georg Wulf

**Affiliations:** ^1^Department of Hematology and Medical Oncology, University Medical Center Goettingen, Goettingen, Germany; ^2^Department of Transfusion Medicine, University Medical Center Goettingen, Goettingen, Germany; ^3^Department of Nephrology and Rheumatology, University Medical Center Goettingen, Goettingen, Germany

**Keywords:** sarcoidosis, hematopoietic stem cell transplantation, HLA, allo and autologous transplantation, immunology

## Abstract

**Purpose:**

Extrinsic factors and genetic predisposition contribute to the etiology of sarcoidosis, converging in a phenotype of altered immune response associated with multisystemic inflammatory granulomatous tissue infiltration. Immunological reconstitution after hematopoietic stem cell transplantation (HSCT) may represent a unique window for the pathogenesis of the disease. We describe the incidence, clinicopathological features, and HLA associations of sarcoidosis after HSCT in a single-center cohort of patients, together with data from previously published cases.

**Methods:**

We retrospectively analyzed clinical characteristics and HLA haplotypes from allogeneic (allo) or autologous (auto) HSCT patients from January 2001 through May 2021 at the University Medicine Goettingen (UMG), and data from previously published cases.

**Results:**

A total number of 19 patients was identified. These included 4 patients from our center (3 allo HSCT and 1 auto HSCT) and 15 patients from the literature review. Thirteen patients had received an allo HSCT, and six patients had received an auto HSCT. Sarcoidosis occurred after a median interval of 20 (after allo HSCT) and 7 (after auto HSCT) months, respectively. The predominant HLA allele associated with sarcoidosis was HLA DRB1*03:01. Sarcoidosis involved the respiratory tract in 15 patients (three unknown, one without pulmonary involvement), and it was associated with graft-versus-host disease in 7 of 13 patients receiving allo HSCT. None of the donors or patients had a history of sarcoidosis before transplantation. Disease manifestations resolved with standard glucocorticoid treatment without long-term sequelae.

**Conclusion:**

Sarcoidosis may occur at low frequency during reconstitution of the immune system after HSCT. HLA allele associations reflect the associations observed in the general population, particularly with DRB1*03:01. Further insights into the interplay between Tcell reconstitution and the development of sarcoidosis could also provide novel approaches to an improved understanding of the pathogenesis in sarcoidosis.

## Introduction

Sarcoidosis is a multisystemic inflammatory disease characterized by noncaseating granulomas, consisting of CD4^+^ T cells and macrophages surrounded by CD8^+^ T cells (1). It frequently affects lungs and lymph nodes but may involve any organ (2). Aberrant interactions of T cells, monocytes, and macrophages after exposure to triggering factors, e.g., (in)organic particles or antigens from infectious agents may contribute to the pathogenic process (2, 3). Genetic susceptibility for sarcoidosis was found associated with distinct human leukocyte antigen (HLA) types, as detected by genome-wide association studies (4, 5). In particular, genetic variants in HLA-DRB1 located in the MHC class II region and the HLA class I antigens A1 and B8 have been associated with an increased risk of disease (6–8).

While DRB1 alleles, such as *03:01 and *03:02, can confer protection against chronic disease (8, 9), HLA DRB1*14 and DRB1*15 are associated with chronic sarcoidosis (2). HLA types implicated in the development of sarcoidosis depending on ethnicity are, among others, HLA-B51 (10), DQB1*02:01 (11), DRB1*03:01 (12, 13), DRB1 *11:01 (13, 14), and DRB1*15:01 (12, 14). An association between sarcoidosis and hematologic malignancies has been observed, documenting sarcoidosis occurring before, during, or after the diagnosis of lymphoma (15–17). Sarcoidosis in the context of hematopoietic stem cell transplantation (HSCT) has been reported rarely (18).

In this study, we determined the incidence and characteristics of sarcoidosis following allogeneic (allo) and autologous (auto) HSCT in a cohort of consecutive patients in the context of findings with previously reported patient data, aiming at a comprehensive clinicopathological view at this rare condition.

## Methods

### Patient Population and Setting

We retrospectively searched our database for sarcoidosis following HSCT in all patients who underwent HSCT between January 2001 and May 2021, with a particular interest in the patients’ and donors’ HLA status and disease manifestations, chemotherapeutic regimens, sarcoidosis manifestations, and overall outcome. The diagnosis of sarcoidosis was assumed based on non-caseating epithelioid granulomas on histologic examination and exclusion of other causes of granulomas. Data retrieved from the literature were reported as stated in the original publications.

### Review of the Literature

Case reports and case series describing sarcoidosis following HSCT were searched using the MeSH terms “stem cell transplantation” and “sarcoidosis” in Medline/Pubmed. Gender, ethnicity, hematologic disease, prior history of sarcoidosis in the donor, remission status before HSCT, donor type, HLA status, conditioning regimen, interval to the onset of sarcoidosis after HSCT, imaging findings, pulmonary function tests (PFTs), involvement of non-pulmonary organs, evidence of graft-versus-host disease (GvHD), and response to glucocorticoids (GC) were extracted from the identified case reports or case series, when available.

### HLA Genotyping

Phenotypes and genotypes are reported as recommended by the WHO Nomenclature Committee for Factors of the HLA System, using four digits in the UMG cohort. HLA phenotypes and genotypes of patients previously reported in the literature are reported as stated in the original publication. Specifically, high-resolution HLA typing was performed by sequence-based typing (SeCore SBT sequencing kits, One Lambda, West Hills, CA, USA) according to the standards for histocompatibility and immunogenetics testing of the European Federation for Immunogenetics (EFI) at our center (19). All 10 HLA loci relevant for transplantation were characterized in the UMG cohort (HLA-A, -B, -C, -DRB1, and -DQB1). Furthermore, HLA-DPB1 was characterized in two out of three UMG patients receiving a matched unrelated donor (MUD) transplantation.

### Statistical Analysis

Data collection and analysis were performed employing Microsoft Excel^®^ (Version 16.48, Microsoft Corporation, Redmond, WA, USA) and GraphPad Prism^®^ Version 9.1 (GraphPad Software, San Diego, CA, USA). Contingency tables and Fisher’s exact test as well as unpaired, two-tailed Student’s *t*-test were used for data analysis, as appropriate. *p*-values <0.05 were considered significant, and *p*-values <0.15 indicate trends worth reporting. Time to onset of disease (cumulative incidence) was calculated using Kaplan–Meier analysis (Log-rank test). The cutoff for age as criterion for the onset of sarcoidosis was calculated using the software X-tile (X-tile, New Haven, Connecticut, USA). Allele frequencies of HLA alleles were calculated *via* direct counting (number of observations for a given allele divided by the number of haplotypes (2 *n*, where *n* = sample size) and the HLA allele frequencies in Germany and Europe (Population 3,6,8) were stated as found in the classical allele frequency search (20).

## Results

**Figure 1:**
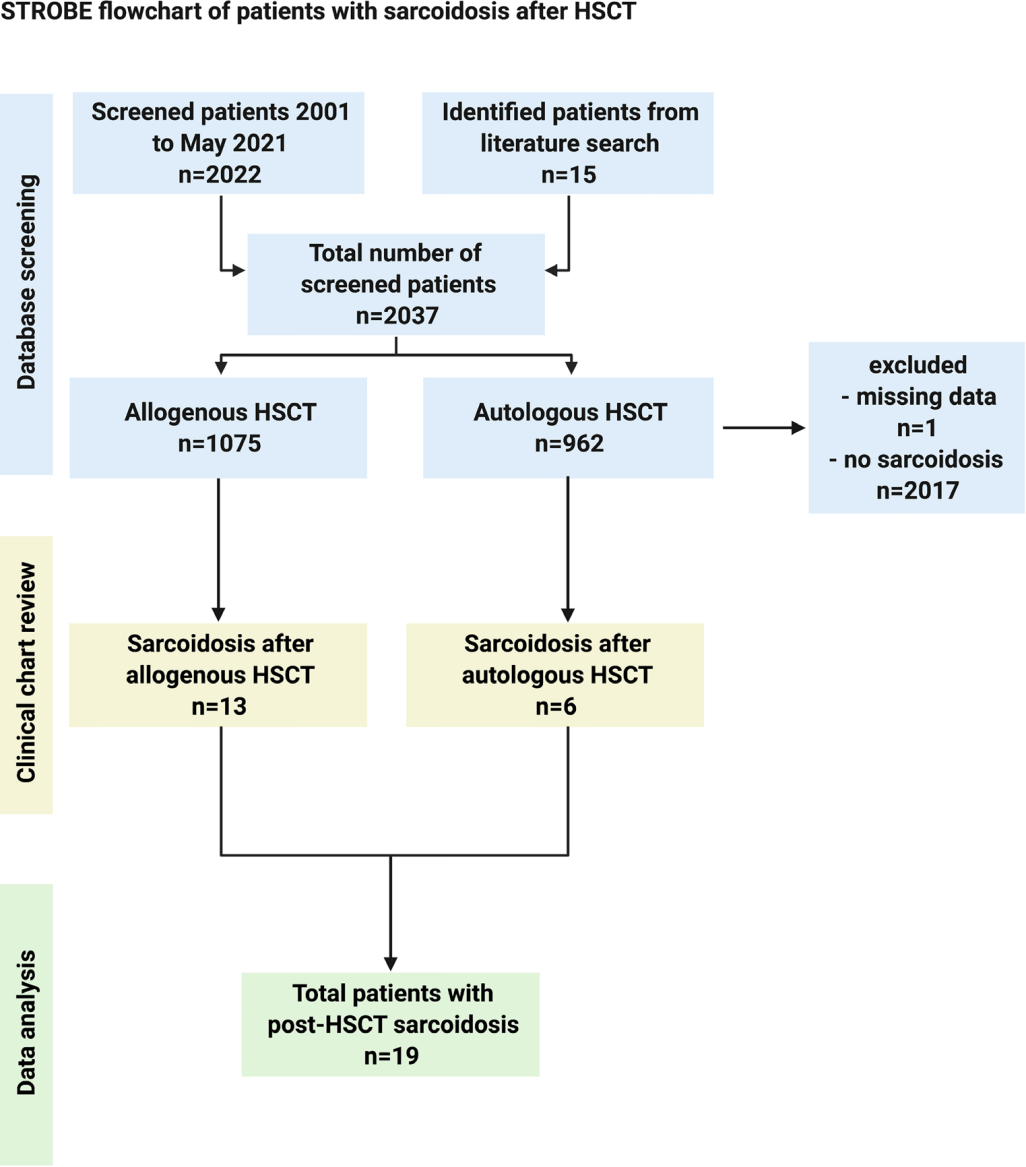
STROBE flowchart of patient disposition.

Of a total number of 2,022 patients who underwent HSCT from 2001 to 2021 at our center, 4 patients who developed sarcoidosis after HSCT were analyzed. An additional 15 patients identified in the literature were included for further analysis. The patient flow is depicted in **Figure 1**.

### Sarcoidosis Following HSCT in Four Patients

Case #1: A 35-year-old male patient without a history of sarcoidosis diagnosed with primary mediastinal B-cell lymphoma received an allo HSCT from a MUD (also without a history of sarcoidosis), leading to a complete remission (CR) without evidence of GvHD. Thirty months later, enlarged pulmonary lymph nodes (LN) and ground glass opacities were noted on chest computed tomography (CT). A LN biopsy showed non-necrotizing granulomas, consistent with a diagnosis of sarcoidosis. Pulmonary function tests showed a reduced diffusion capacity for carbon monoxide (DLCO/SB) and forced vital capacity (FVC), compatible with a restrictive pattern. As the patient remained asymptomatic, no specific treatment was initiated. A subsequent CT showed spontaneous regression of the pulmonary changes after six months.

Case #2: A 65-year-old male patient diagnosed with angioimmunoblastic T-cell lymphoma (AITL) received an allo HSCT from a MUD with no known history of sarcoidosis leading to a CR without evidence of GvHD. Eighteen months after HSCT, the patient reported dyspnea and had a restrictive pattern on PFT. A chest CT showed basal consolidations and enlarged mediastinal LN. A bronchoalveolar lavage (BAL) showed non-specific inflammation without findings suggestive of sarcoidosis. Two months later, non-caseating granulomas consistent with sarcoidosis were histologically diagnosed after biopsy of a pulmonary lesion, and the patient was started on GC therapy with a tapering regimen, eliciting a good clinical response.

Case #3: A 56-year-old male patient diagnosed with acute myelogenous leukemia (M5) received an allo HSCT from a matched related donor (MRD) without a history of sarcoidosis. Subsequently, the patient suffered from cutaneous, hepatic, and intestinal GvHD. Twenty-four months after allo HSCT, the patient complained of acute abdominal and thoracic pain, which was interpreted as post-herpetic neuralgia and side effects of gastrointestinal GvHD. A chest and abdominal CT showed bilateral hilar lymphadenopathy, intrapulmonary nodules, and prominent abdominal lymph nodes. Sarcoidosis was diagnosed following biopsy of a hilar LN, and, as the patient was asymptomatic, a watch-and-wait strategy was employed. However, in the following months, the patient complained of arthralgias with shoulder pain, morning stiffness in the fingers and elbows, as well as shortness of breath following moderate physical exertion. Therefore, a GC regimen with concomitant non-steroidal antirheumatic drugs was initiated with good clinical regression of symptoms.

Case #4: A 56-year-old male patient was diagnosed with AITL. Standard chemotherapy was consolidated with auto HSCT. Five months after transplantation, enlarged pulmonary LN were noted on routine chest CT, and histopathologic examination showed granulomas consistent with pulmonary sarcoidosis. Oral GC therapy was initiated leading to a remission of pulmonary nodules and LN. After tapering of GC, an increase in size of mediastinal and infradiaphragmal LN was noted, and a repeat biopsy was performed leading to the diagnosis of a relapse of the AITL. An allo HSCT from a MRD with no known history of sarcoidosis was performed. Post allo HSCT, the patient suffered from GC-sensitive cutaneous and intestinal GvHD; no relapse of sarcoidosis was noted.

### Patient Characteristics

Overall, we observed symptomatic sarcoidosis in a total of 4 out of 1,065 and 957 patients, who had received allogeneic and autologous stem cell transplantations in the UMG transplantation program, respectively. The disease emerged within a median follow-up observation period of 22 months, resulting in an estimated incidence of 156/100,000 and 58/100,000 for patients at risk after allo and auto HSCT, respectively.

An additional 15 cases of sarcoidosis following HSCT have been reported in the literature, with 5 patients having received an auto HSCT (18, 21, 22), while 10 had received an allo HSCT (18, 23–30). Together with our patients, this translates into a total number of 19 reported cases. Although the total populations of the previously published case reports are unknown, the overall low incidences in the allogeneic transplantation group appear consistent between our cohort and the published data. The patient characteristics are shown in **Tables 1** and **2**. At our center, we also identified 11 patients with a previous history of clinically apparent sarcoidosis prior to HSCT. None of these patients developed histologically proven symptomatic sarcoidosis following HSCT. Of note, only four patients with sarcoidosis prior to HSCT could be observed beyond the median time of disease emergence of 22 months, with a median post-transplantation observation period of 52 months.

**Table 1:** Summary of sarcoidosis patient characteristics after allogenous HSCT.

Parameter	Patient number												
	#1UMG cohort	#2UMG cohort	#3UMG cohort	#4 (23)	#5 (24)	#6 (25)	#7 (26)	#8 (18)	#9 (27)	#10 (27)	#11 (28)	#12 (29)	#13 (30)
Age*/gender	39 M	69 M	59 M	34 M	38 F	37 F	55 F	52 F	46 F	52 M	48 M	65 F	58 F
**Ethnicity**	C	C	C	UKN	UKN	UKN	A	C	C	C	C	A	UKN
**Diagnosis**	PMBL	AITL	AML	NHL	LBL	CML	FL	CML	MDS	MDS	CMML	ATL	MF
**History of sarcoidosis in donor**	N	N	N	Y	Y	Y	N	UKN	UKN	N	N	N	N
**Remission status prior to HSCT**	PR	CR	CR	UKN	UKN	UKN	CR	Relapse	UKN	UKN	MR	UKN	UKN
**Donor type**	MUD	MUD	MRD	MRD	MRD	MRD	MUD	UKN	MUD	MUD	MRD	MUD	MRD
**HLA status^#^**	A: 0201 B: 1501,4001 C: 0304 **DR:** **0301**, 0901 **DQ:** **0201**,0303 DP: 0401	A: 0101,0201 **B**: 4002,**5101** C: 0202,1502 **DR:** **1101**; DQ: 0301 DP: 0401	A*: 01:01/. B* 07:02/ **08:01** C* 07:01/ 07:02 DRB1* **03:01**/ 15:01 DQB1* 02:01/ 06:02	A1, A2, **B8, B51**, DR4, DR4	A(1,26); B(8,49); Bw(4,6); Cw (7,-); DRß1*03011; DRß1*1302	A-24(9) **B-8** 52(5) C-7 16; DR-17(3) 18(3) DQ-2 4 DP-	UKN	UKN	UKN	UKN	A*0101, A*2501, B*0801, B*1801, DRB1*0301, DRB1*1501, DQB1*0201, DQB1*0602	UKN	A*0301, B*0702, B*5501, C*0702, C*0304, DRB1* 1301, DRB1* 1501, DQB1*0602
**Conditioning regimen**	FLU BU CYC -ATG	FLU BU CYC -ATG	FLU BU	UKN	TBI, Eto	TBI, CYC	TBI, FLU BU	TBI, CYC	FLU BU Alem	FLU BU Alem	BU CP	TBI, FLU BU	UKN
**Sarcoidosis DX post HCT (months)**	30	20	24	3	17	21	6	6	12	20	22	16	27
**Chest CT/radiographic changes**	Y	Y	Y	Y	Y	UKN	Y	Y	Y	N	Y	UKN	UKN
**Abnormal PFT**	Y	Y	UKN	UKN	UKN	UKN	UKN	Y	UKN	UKN	UKN	UKN	UKN
**Non-pulmonary organs involved**	N	N	MSK	Liver	N	Bone marrow	N	N	Skin, Liver	Liver	Skin	Skin	Skin
**Evidence of GVHD**	N	N	Y	Y	Y	N	N	Y	UKN	UKN	Y	Y	Y
**Response to GC**	UKN	Y	Y	Y	Y	Resolution after DLI	Asymp, nt	Y	Y	Y	Y	Asymp, nt	UKN

A, Asian; AA, African American; AITL, angioimmunoblastic T-cell lymphoma; Alem, alemtuzumab; AML, acute myelogenous leukemia; Asymp, asymptomatic; ATG, anti-thymocyte globulin; ATL, adult T-cell leukemia; BU, busulfan; C, Caucasian; CML, chronic myelogenous leukemia; CMML, chronic myelomonocytic leukemia; CR, complete remission; CYC, cyclophosphamide; DLCO, diffusion capacity for carbon monoxide; DLI, donor lymphpocyte infusion; Dx, diagnosis; Eto, etoposide; F, female; FL, follicular lymphoma; FLU, fludarabine; GC, glucocorticoids; HLA, human leukocyte antigen; LBL, large B-cell lymphoma; M, male; MDS, myelodysplastic syndrome; MF, myelofibrosis; MR, minimal response; MRD, matched related donor; MSK, musculoskeletal system; MUD, matched unrelated donor; N, no/none; NHL, non-Hodgkin lymphoma; nt, no treatment; PFTs, pulmonary function tests; PMBL, primary mediastinal B-cell lymphoma; PR, partial remission; TBI, total body irradiation; UKN, unknown; VC, vital capacity; Y, yes. *Age at diagnosis of sarcoidosis; ^#^HLA status with known association to sarcoidosis reported in bold.

**Table 2:** Summary of sarcoidosis patient characteristics after autologous HSCT.

Parameter	Patient number					
	#1UMG cohort	#2 (18)	#3 (18)	#4 (18)	#5	#6 (22)
Age*/gender	60 M	50 F	47 F	48 F	62 F	25 M
**Ethnicity**	C	AA	C	C	UKN	UKN
**Diagnosis**	AITL	Breast	Breast	Breast	POEMS	Testicular Cancer
**Remission status prior to HSCT**	PD	UKN	UKN	UKN	PD	PD
**Conditioning regimen**	HD-BEAM	UKN	UKN	UKN	Melphalan	﻿Carboplatin and etoposide
**Sarcoidosis DX post HCT (months)**	5	8	3	120	6	60
**Chest CT/radiographic changes**	Y	Y	Y	Y	Y	Y
**Abnormal PFT**	UKN	Y	UKN	UKN	UKN	UKN
**Extrapulmonary organs involved**	N	MSK	Skin	N	LN	N
**Response to GC**	Y	Y	Y	Asymp, nt	Asymp, nt	Y

AA, African American; AITL, angioimmunoblastic T-cell lymphoma; Asymp, asymptomatic; C, Caucasian; F, female; GC, glucocorticoids; LN, lymph nodes; M, male; MSK, musculoskeletal system; N, no; PD, progressive disease; POEMS, polyneuropathy, organomegaly, endocrinopathy, monoclonal protein, skin changes syndrome; UKN, unknown; Y, yes. *Age at diagnosis of sarcoidosis.

### Underlying Disease in 19 Patients Who Developed Sarcoidosis After Stem Cell Transplantation

The most frequently reported underlying disease leading to treatment with allo HSCT was lymphoma (*n* = 7) followed by leukemia (*n* = 4) and myelodysplastic syndrome (MDS)/myelofibrosis (MF) (*n* = 3). The reported underlying diseases leading to auto HSCT were breast or testicular cancer (*n* = 4), as well as polyneuropathy, organomegaly, endocrinopathy, monoclonal protein, skin changes (POEMS) syndrome in one patient.

### Donor Status

Of the 13 patients who received an allo HSCT, 6 patients were transplanted from a MUD, 6 from a MRD, and in 1 case, the donor status was not reported. Five patients received peripheral blood stem cell donations, five received bone marrow transplant donations, and in three patients, the mode of transplantation was not specified. None of the three donors of our cohort had a history of sarcoidosis, while this was the case in 3 of 10 donors in the cohort of published case studies (23–25).

### Types of Chemotherapy

The most frequently applied chemotherapeutic agents in the allo HSCT cohort were busulfan (BU) (*n* = 8), fludarabine (FLU) (*n* = 7), and cyclophosphamide (CYC) (*n* = 5). Two patients each also received alemtuzumab (ALM) or antithymocyte globulin (ATG) as part of the conditioning regimen, respectively. In five patients, chemotherapy had been combined with total body irradiation (TBI). In the autologous cohort carboplatin, etoposide, BEAM, and melphalan were the most frequently applied cytostatic drugs.

### Clinical Characteristics

All patients had pulmonary sarcoidosis. Most patients showed changes on CT/chest radiographs (15/19; *n* = 3 unknown [UKN], *n* = 1 no changes). Involvement of extrapulmonary organs was present in 11/19 patients; the most common organ manifestation was skin followed by liver. Seven of the 13 allo HSCT also had evidence of GvHD; in two patients, the GvHD status was not reported. A response to GC was noted in all patients where they were given (12/19; 2 UKN, 4 asymptomatic, 1 resolution of symptoms after donor lymphocyte infusion [DLI]). No additional immunosuppressive therapies were used. The characteristics of the patients in both groups (allo vs. auto HSCT) are summarized in **Table 3**.

**Table 3:** Comparison of general patient characteristics in the allogeneic and autologous transplantation group.

Parameter	Allogeneic HSCT *n* = 13	Autologous HSCT *n* = 6
**Age**, median (range)	52 (34–69)	49 (25–62)
**Female sex**, *n* (%)	7 (53.8%)	4 (66.7%)
**Ethnicity**, *n* (%) - Caucasian - African American - Asian - Not reported	7 (53.8%) 0 2 (15.4%) 4 (30.8%)	3 (50%) 1 (16.7%) 0 2 (33.3%)
**Median time to sarcoidosis after HSCT** (months)	20 (3–30)	7 (3–120)
**Sarcoidosis manifestations**, *n* (%) - Lung - Skin - Liver - MSK - Bone marrow - Lymph node	13 (100%) 4 (30.8%) 3 (23.1%) 1 (7.7%) 1 (7.7%) 0	6 (100%) 1 (16.7%) 0 1 (16.7%) 0 1 (16.7%)
**Treatment** - Glucocorticoids, *n* (%) - No treatment, *n* (%) - Not reported, *n* (%) - Other treatment, *n* (%)	8 (61.5%) 2 (15.4%) 2 (15.4%) 1 (7.7%)	4 (66.7%) 2 (33.3%) 0 0
**Response to treatment** [of those treated, *n* (%)]	9 (100%)	4 (100%)

HSCT, hematopoietic stem cell transplantation; MSK, musculoskeletal; n, number.

### HLA Genotypes in Association With Sarcoidosis and HSCT

**Figure 2:**
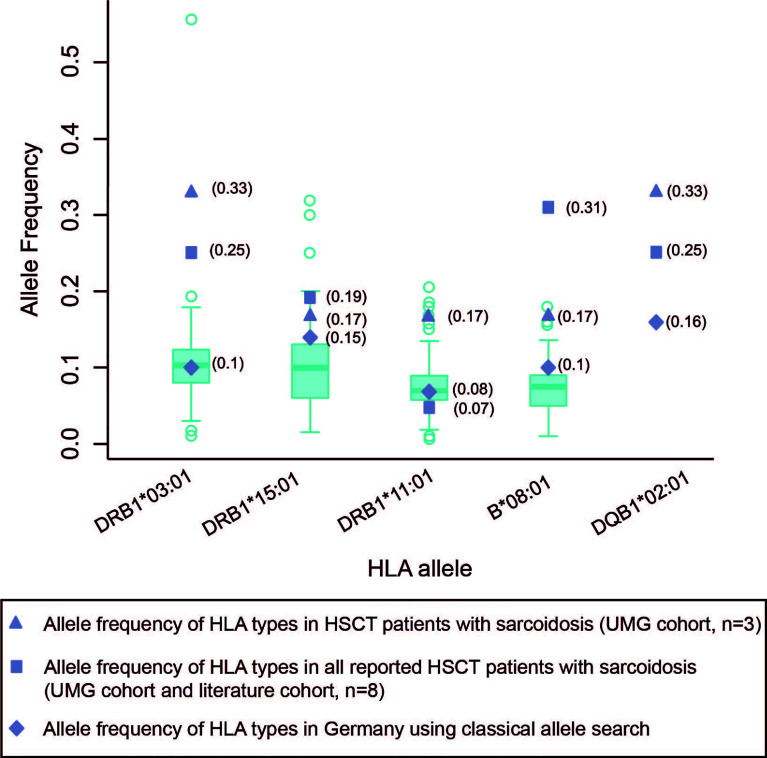
Allele frequency of HLA types in HSCT patients with sarcoidosis compared to German and European normal population cohorts. Box plots of HLA allele frequencies in Europe shown where available (20). The calculation of allele frequency in our cohort and published case report cohorts were obtained *via* direct counting (number of observations for a given allele divided by the number of haplotypes [2 *n*, where *n* = sample size]). UMG, University Medical Center Göttingen.

The HLA status was reported in eight cases. Four of these patients expressed the DRB1*03:01 HLA allele, already known for a strong association with sarcoidosis. Similarly, all other patients also exhibited HLA types known to be associated with sarcoidosis (**Tables 1** and **2**). Specifically, the allele frequencies of DRB1*03:01 and DQB1*02:01 were higher in our cohort compared to the respective allele frequency in the cohort of all reported patients with sarcoidosis following HSCT (our cohort and published case reports). The allele frequencies for the respective genotypes in this cohort were, in turn, higher than the allele frequency reported in a German and European reference population (20) (**Figure 2**).

### Cumulative Incidence of Post-HSCT Sarcoidosis

**Figure 3:**
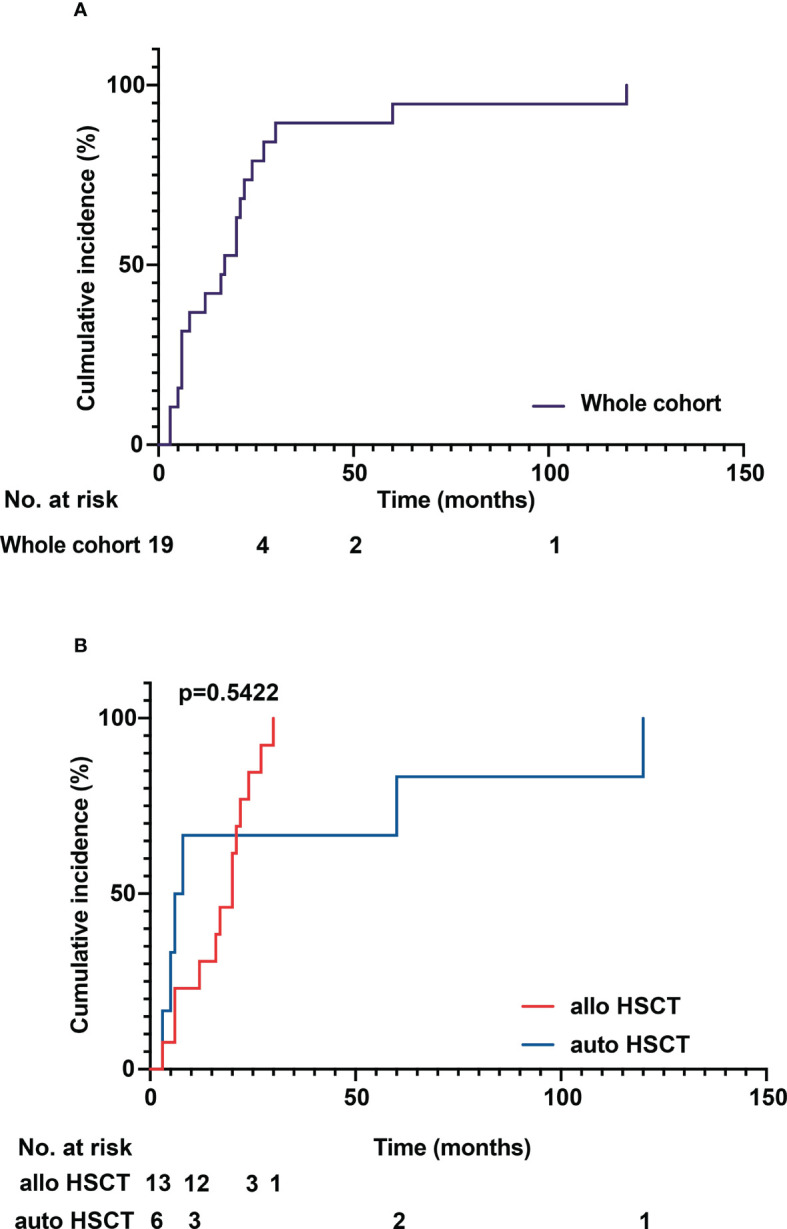
**(A)** Cumulative incidence of sarcoidosis over time in the whole cohort. **(B)** Cumulative incidence of sarcoidosis in allogenous HSCT versus autologous HSCT. The median time to onset of sarcoidosis post HSCT overall was 17 months (range 3 to 120 months), with patients developing sarcoidosis after allo HSCT at a median time of 20 months (range 3 to 30 months) and 7 months (range 3 to 120 months) after auto HSCT. No statistically significant difference between the groups could be detected (*p* = 0.5422).

**Figure 4:**
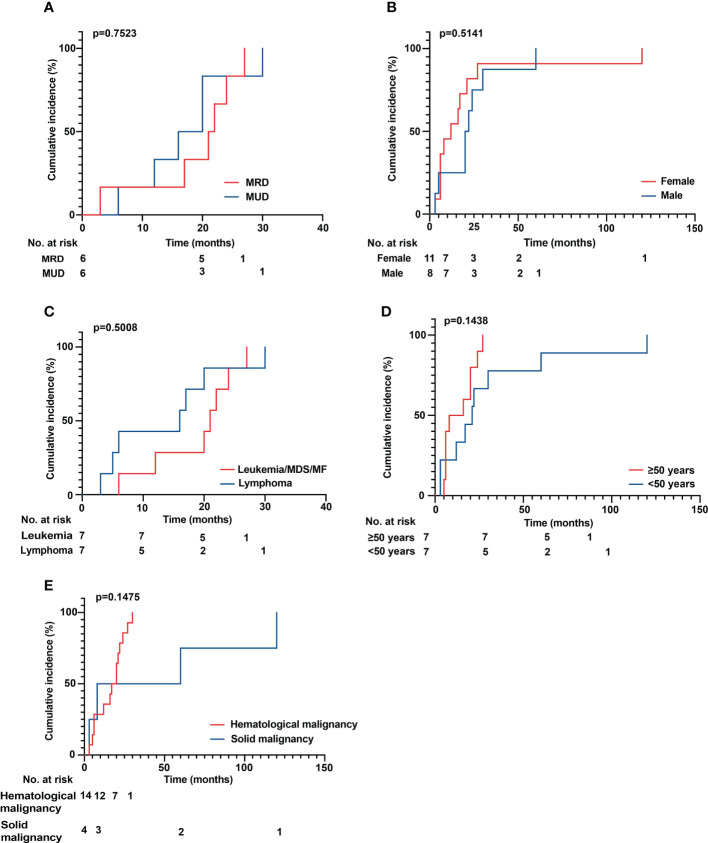
**(A–E)** Cumulative incidence of sarcoidosis over time. **(A)** Comparison of MRD versus MUD donor status. **(B)** Male versus female patients. **(C)** Leukemia versus lymphoma/MDS/MF. **(D)** Comparison of patients aged older than 50 years versus younger than 50 years. **(E)** Comparison of hematological versus solid malignancies. No statistically significant differences could be detected. MDS, myelodysplastic syndrome; MF, myelofibrosis; MRD, matched related donor; MUD, matched unrelated donor.

The median age of patients with sarcoidosis after HSCT was 50 years (range 25 to 69 years). The median time to onset of sarcoidosis post HSCT overall was 17 months (range 3 to 120 months), with patients developing sarcoidosis after allo HSCT at a median time of 20 months (range 3 to 30 months) and 7 months (range 3 to 120 months) after auto HSCT (*p* = 0.5422) (**Figure 3A**). In patients over 50 years of age, the median time to sarcoidosis after HSCT was 12 months, while it was 21 months in patients younger than 50 years (*p* = 0.1438) (**Figure 3B**). Overall, patients were more often female (11 vs. 8) and of Caucasian ethnicity. There were no statistically significant differences between groups. Furthermore, we analyzed the cumulative incidence of sarcoidosis depending on gender, donor status (MRD/MUD), solid vs. hematological neoplasia, and lymphoma vs. leukemia/MDS/MF (**Figures 4A–E**). Due to the low overall numbers, no statistically significant differences were detected.

## Discussion

Symptomatic sarcoidosis after HSCT represents a rare condition, and we here provide an estimate of incidences in the autologous and allogeneic transplantation setting and its clinical characteristics. Sarcoidosis post HSCT occurred in patients receiving both allogeneic and autologous stem cell transplantations at a ratio of 3:1, both in the UMG and the previously published patients. The increased frequency of sarcoidosis after allogeneic transplantation is compatible with the strong activation of the immune system upon engraftment in these patients. This observation suggests that a preformed immunological reaction may occur with increasing immunological competence of the Tcell repertoire and/or antigen-presenting cells (APCs). This notion is further supported by the HLA allele associations observed for sarcoidosis post HSCT, which reiterates the associations described for sporadic sarcoidosis (2, 8–14) (**Table 1**). While the antigen(s) triggering sarcoidosis remain elusive, our observations were best compatible with a minor self-antigen or a preexisting viral antigen, which is presented to the newly engrafted immune system in an HLA allele associated efficacy to elicit the sarcoidosis-type reaction in the post transplantation period (median time of 17 months, 20 months after allo HSCT, and 7 months after auto HSCT).

Seven of 13 patients with sarcoidosis post allogeneic HSCT also developed acute or chronic GvHD in the post-transplantation follow-up. Dependent on the precise transplantation setting, approximately 60% of patients develop GvHD after allo HSCT. Thus, our data do not support an association of sarcoidosis and GvHD, albeit this is limited by the small patient numbers.

Immunologically, granuloma formation is initiated by an interaction between CD4+ T cells and APCs, such as macrophages or dendritic cells *via* HLA class II antigen–peptide complex leading to T-cell activation, differentiation into Th1 cells, secretion of interleukin (IL)-2 and interferon (IFN)-g, and augmented macrophage tumor necrosis factor (TNF)-a production, resulting in immune response amplification (31–33). After granuloma formation, there are two possible outcomes: Granuloma formation can either resolve, if the peptide antigens are presented by HLA-DR3 molecules on dendritic cells or macrophages and subsequently recognized by T cells leading to the release of a range of cytokines, or persistent granulomatous inflammation with subsequent tissue damage requiring immunosuppressive therapy (32). The latter is thought to occur if the antigen recognition is inefficient and can be due to peptides displayed by molecules other than HLA-DRB1*03 (HLA-DR3) or T cells that are not capable of generating efficient T-cell clones (34).

Two agents that interfere with both Tcell and- dendritic cell (DC)-mediated immunity, which is necessary for the resolution of sarcoid granulomas, are ATG and ALM. Two of our allo HSCT patients received ATG, while it was not reported in any of the case reports. ATG, a polyclonal antibody, is used to prevent GvHD and suppress allograft rejection. Owing to its polyclonality, there are diverse effects of ATG on the immune system, among them Tcell depletion, modulation of leukocyte/endothelial interactions, apoptosis in B cells, interference with DC function and the induction of regulatory T and natural killer (NK) T cells (35). So far there have been no reported cases linking sarcoidosis to ATG treatment.

Alemtuzumab, however, has been associated with the development of sarcoidosis and there are several case reports describing the occurrence of sarcoidosis post ALM treatment in multiple sclerosis patients (36–38). While the exact mechanism by which ALM triggers sarcoidosis is not yet known, a dysregulation in the Th1/Tc1 cell/IFN-γ network and the development of autoantibodies by dysregulated B cells has been postulated (39). The immune dysregulatory effects of ATG and ALM could play a role in the development of sarcoidosis in the post HSCT setting; however, taking into account the heterogenous usage of ATG and ALM in the reported patients, no clear causal link can be inferred.

Different hypotheses have been proposed, aiming to explain the pathogenesis of sarcoidosis after malignancy: Firstly, it has been postulated that sarcoidosis develops as a reaction to the immunosuppressive effect of chemotherapies or to a specific chemotherapy agent, such as bleomycin, known to achieve high concentrations in LN, skin, and lung tissue (40).

Secondly, Brincker et al. hypothesized that the appearance of sarcoidosis post malignancy may be driven by antigens derived from tumor cells leading to immunological events resulting in granuloma formation (41). Thirdly, Kornacker et al. suggested that underlying immunologic disturbances associated with the primary malignancy may lead to the formation of epithelioid granulomas (42).

In three patients the occurrence of sarcoidosis has been reported after possible transmission from the donor (23–25). The data from our patient cohort do not support an increased risk of transmission, as none of our patients was transplanted from a donor with a previous history of sarcoidosis, and *vice versa*, none of the patients with sarcoidosis in their pre-transplant medical history experienced sarcoidosis recurrence.

**Figure 5:**
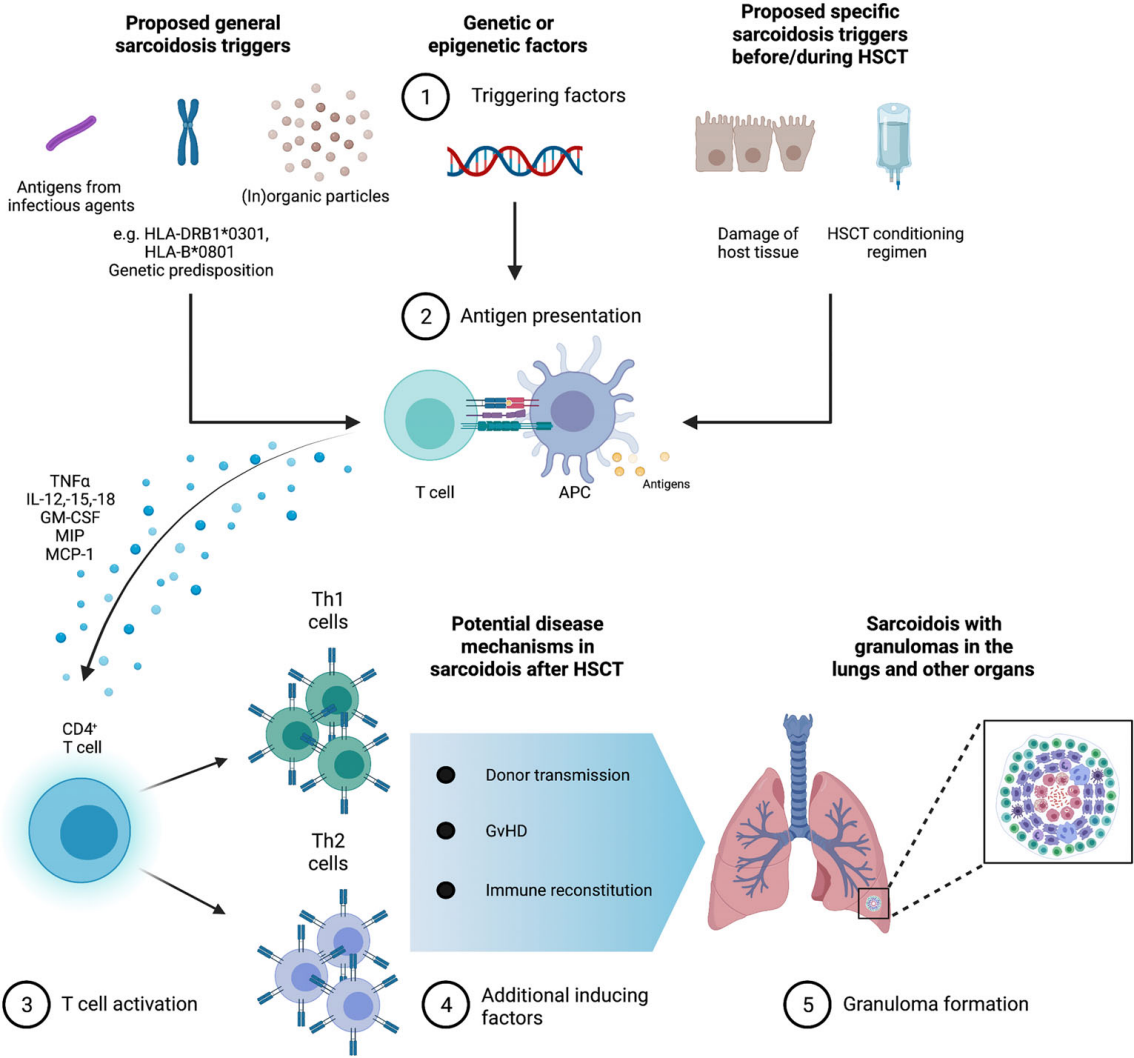
Granuloma formation in sarcoidosis with possible triggers in the context of hematopoietic stem cell transplantation (HSCT). Infectious, organic, and inorganic agents are possible triggers for sarcoidosis against the background of genetic and environmental factors. Furthermore, specific sarcoidosis triggers in HSCT could be the HSCT conditioning regimen and disease damage of host tissues. Antigen-presenting cells (APC) produce high levels of TNF α, interleukins (IL)-12, -15, and -18, macrophage inflammatory protein 1 (MIP-1), monocyte chemotactic protein 1 (MCP-1), and granulocyte macrophage colony-stimulating factor (GM-CSF). CD4+ T cells initiate differentiation into Th1 helper cells that secrete predominantly interleukin-2 and interferon gamma IFN-γ as well as Th2 helper cells that secrete IL-4, -5, -6, and -10 stimulate fibroblast proliferation and collagen production leading to the formation of granuloma and possible fibrosis. In the context of HSCT, granuloma formation could be enhanced by graft versus host disease (GvHD), immune reconstitution, and donor transmission.

The immunological environment post HSCT, similar to that present in sarcoidosis pathogenesis outside of the context of HSCT, could promote the development of sarcoidosis, especially in a genetically susceptible individual with a specific HLA allele. The reported disease pathogenesis and potentially triggering factors are summarized in **Figure 5**: Before HSCT, the conditioning regimen as well as underlying disease damage of host tissues induce pro-inflammatory cytokines such as TNF-a, chemokines, and costimulatory molecules on host APCs. Following HSCT, donor T cells proliferate and differentiate in response to activated host APCs, then expressing IFN-g, IL-2, and TNF-a, leading to T-cell expansion and differentiation into Th1 vs. Th2 subtypes (43, 44). This is followed by an increase in the number of suppressor/cytotoxic lymphocytes and then of helper–inducer phenotype T cells, thereby inverting CD4+/CD8+ ratios. A normalization of CD4+/CD8+ T cell ratios takes place approximately 1 to 2 years after HSCT (45, 46) with a longer time span in older patients, whose thymic function is less pronounced.

During immune reconstitution, higher levels of cytokines such as MCP-1, CCR1, CCR2, IL-8, and Rantes are present, leading to a tissue environment promoting the formation of non-necrotizing epithelioid granulomas (47–49).

All allogenously transplanted patients reported previously, including those in our cohort, developed sarcoidosis after complete donor engraftment of their bone marrow, suggesting that the development of sarcoidosis in these circumstances was initiated by the donor immune system. Interestingly, it has been shown that individuals with a HLA-B8/DR3 phenotype produce higher amounts of TNF-a compared to their IFN-g generation potential (50), making them susceptible to humoral hyperreactivity and anergy under an environmental stress. Overall, the time course of sarcoidosis development in the described patients within this report (ranging from three to 120 months after HSCT) is consistent with what would be expected from the current hypothesized mechanisms of granuloma formation, which usually take months rather days or weeks to form (51).

The older age and shorter time to the onset of sarcoidosis post HSCT of the analyzed patients compared to the normal population might be explained with the progressive involution of thymic tissue during aging with a decline in T-cell output and T-cell senescence with restricted T-cell receptor repertoire diversity, leading to a slower immune reconstitution and impaired immune responses following transplantation (46). However, a bias that patients with malignancies, especially lymphomas, are generally older must be considered.

The occurrence of extrapulmonary manifestations of sarcoidosis in patients with leukemia, MDS, and MF compared to lymphoma patients has been linked to malignant antigens as a sarcoidosis trigger in leukemic diseases (41). While the remission status prior to HSCT of most reported patients in the literature is unknown, sarcoidosis in our cohort patients occurred exclusively in patients with complete disease remissions, rendering a malignant antigenic trigger for sarcoidosis unlikely. Specific chemotherapeutic agents, such as bleomycin, have been hypothesized to trigger sarcoidosis. Interestingly, the majority of the allo HSCT patients received a regimen including BU, FLU, and CYC. There have been no reports implicating FLU and BU in the formation of sarcoidosis, CYC has been used previously in the treatment of severe cardiac or neurosarcoidosis (52). TBI is known to lead to a delayed immune reconstitution (46).

Our study has several limitations. First, sarcoidosis post HSCT is a rarely reported event, although based on observations by Bhagat et al. (18) and the prevalence in our cohort of HSCT patients, the occurrence in HSCT might be higher than previously estimated. Also, the number of patients with accesible data for analysis was limited and the small number of cases may influence trends we observed in sub-groups during data analysis. Furthermore, multicentric analyses in additional cohorts may gain more granular insights into the prevalence, potential triggers, and the pathogenesis of sarcoidosis post HSCT. Lastly, it has to be noted that the diagnosis of sarcoidosis can never be ascertained based on granulomas alone, but, at least in our patients, we are confident to have excluded alternative reasons for granulomatous reaction. For the patients in the literature, we had to rely on the reported data. Nevertheless, our report has several strengths: To our knowledge, this report represents the most comprehensive analysis from a single center in conjunction with previously published data. The strong HLA association reported and corroborated by our data suggest that sarcoidosis occurrence in this vulnerable population is influenced by a genetic predisposition requiring additional immunological events in the context of HSCT.

## Conclusions

Based on the 2022 HSCT patients that have been treated at our institution over a 20-year span and four cases of sarcoidosis occurring therein, we estimate the incidence at 156 and 58 cases per 100,000 for allo and auto HSCT, respectively, which is higher than reported by Bhagat et al. (18) and higher than reported in the general German population (53). Based on the low number of incident cases, this is, however, only a rough estimate. Overall, sarcoidosis after allo or auto HSCT is a rare event, frequently affects the lungs and skin, but usually responds to GC treatment. Further insights into the interplay between T cell reconstitution and the formation of sarcoidosis may also provide insight into the overall pathogenesis of sarcoidosis.

## Data Availability Statement

The original contributions presented in the study are included in the article/supplementary material. Further inquiries can be directed to the corresponding authors.

## Ethics Statement

The studies involving human participants were reviewed and approved by the Ethics Committee of the University Medical Center Göttingen (protocol number 30/6/21). Written informed consent for participation was not required for this study in accordance with the national legislation and the institutional requirements.

## Author Contributions

RW-K and PK designed the study. Material preparation, data collection, and analysis were performed by RW-K, JB, JK, JGR, GW, and PK. JH and WJ treated patients and edited the manuscript. The figures were created by RW-K and PK. The first draft of the manuscript was written by RW-K. All authors contributed to the article and approved the submitted version.

## Funding

We acknowledge support by the Open Access Publication Funds of the Göttingen University.

## Conflict of Interest

The authors declare that the research was conducted in the absence of any commercial or financial relationships that could be construed as a potential conflict of interest.

## Publisher’s Note

All claims expressed in this article are solely those of the authors and do not necessarily represent those of their affiliated organizations, or those of the publisher, the editors and the reviewers. Any product that may be evaluated in this article, or claim that may be made by its manufacturer, is not guaranteed or endorsed by the publisher.
